# Synergistic Integration of Enzyme and Microbial Platforms for Sustainable Management of Pharmaceutical Pollutants: Towards a Greener Pharmaceutical Lifecycle

**DOI:** 10.3390/biology15100804

**Published:** 2026-05-19

**Authors:** Zhongshan Sun, Peitao Chen, Xiangyang Ge, Weiguo Zhang, Huanmin Liu

**Affiliations:** 1 School of Pharmacy & School of Biological and Food Engineering, Changzhou University, Changzhou 213164, China; 2200390223@smail.cczu.edu.cn (Z.S.); 2200390214@smail.cczu.edu.cn (P.C.); 2 School of Biotechnology, Jiangnan University, Wuxi 214126, China

**Keywords:** enzyme, microorganism, synergistic integration, pharmaceutical pollutants, sustainable management, green pharmaceuticals

## Abstract

**Purpose:** This review aims to provide a theoretical basis and scientific reference for constructing environmentally friendly and economically feasible sustainable management systems for pharmaceutical pollution. **Methods:** This review discusses three synergistic mechanisms—“cascade degradation”, “symbiotic protection”, and “functional complementarity”—along with construction strategies including co-immobilization technology, engineered biofilms, and engineered bacteria modified via synthetic biology. **Result:** Synergistic platforms have achieved significant progress in treating various types of pharmaceutical pollutants, including antibiotics, anti-inflammatories and hormones, antiviral drugs and pesticides. **Conclusions:** The synergistic integration of enzymes and microorganisms achieves the unification of efficient catalysis and deep mineralization, opening up a new pathway for the remediation of pharmaceutical pollution. It also transforms theoretically existing concepts into operable treatment technologies.

## Simple Summary

As pharmaceutically active compounds continuously accumulate in the environment, pharmaceutical pollutants have emerged as a significant concern threatening ecosystems and public health. Conventional physicochemical methods exhibit numerous shortcomings when dealing with such structurally complex trace pollutants: high treatment costs, difficulty in avoiding secondary pollution risks, and often unsatisfactory degradation efficiency. Single biotechnology approaches, whether enzymatic catalysis or microbial degradation, are similarly constrained by inherent limitations—the former suffers from poor stability, while the latter has a narrow substrate spectrum. Therefore, integrating the high-efficiency catalytic properties of enzymes with the metabolic diversity of microorganisms to construct synergistic treatment platforms has become a critical pathway to overcome these challenges. This review focuses on enzyme–microbe synergistic systems, systematically analyzing the practical dilemmas in pharmaceutical pollution control. It provides an in-depth exposition of the latest advances in the three major synergistic mechanisms and three construction strategies for the treatment of typical pharmaceutical contaminants.

## 1. Dilemmas in Pharmaceutical Pollution Control: From Conventional Shortcomings to the Inevitability of Biological Synergy

### 1.1. Environmental Fate and Ecological Risks of Pharmaceutical Pollutants

As emerging environmental contaminants, pharmaceutical pollutants have become a global challenge due to their widespread occurrence and ecological impacts. With the rapid development of the pharmaceutical industry and population growth, pharmaceutical consumption continues to rise, leading to pharmaceutically active compounds entering the environment through multiple pathways. Industrial wastewater discharge during production, human and animal excreta during consumption, improper disposal of expired medications, and direct use in aquaculture and animal husbandry constitute the main sources of pharmaceutical pollution [1,2].

**Figure 1:**
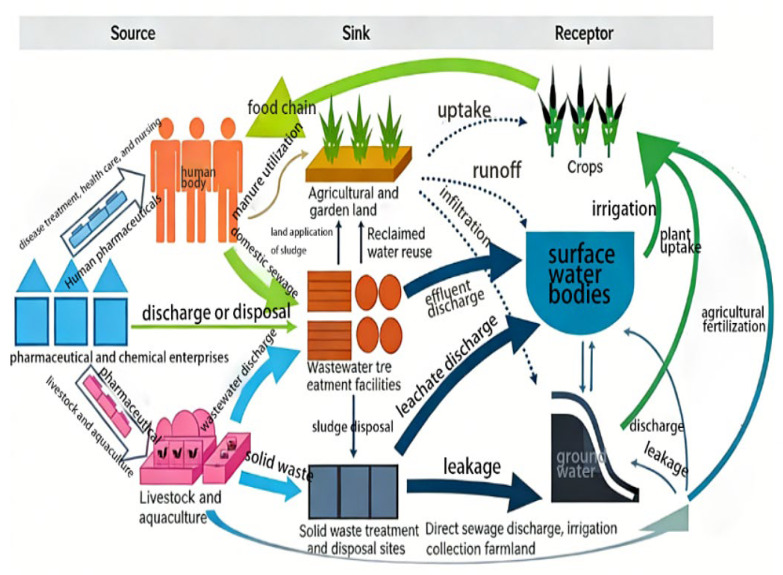
Schematic diagram of the environmental sources, fate, and ecological risks of pharmaceutical pollutants.

Pharmaceutical pollutants are widely detected in water bodies, soil, and sediments. Although their concentrations are typically at trace levels (ng/L to μg/L), continuous input and “pseudo-persistence” characteristics make their ecological risks non-negligible. Antibiotics are particularly concerning, with ciprofloxacin, sulfamethoxazole, and tetracycline frequently detected in environmental samples from different regions [3,4,5]. For example, studies in East Africa show that the risk quotient (RQ) for ciprofloxacin in Kenyan water bodies ranged from 3.5 to 40.6, and 0.1–3.53 for sulfamethoxazole [6], while the RQ for ciprofloxacin in Ethiopia also reaches 8.58. More critically, pharmaceutical pollution is closely linked to the spread of antibiotic resistance—the widespread presence of antibiotic resistance genes in environmental samples has been confirmed to be directly associated with pharmaceutical pollution [7,8]. Studies on Chinese pig farms show diverse and abundant antibiotic resistance genes detected in fecal samples [9], and the evolution of resistance genes during sewage sludge composting is closely related to bacterial community dynamics [10] (Figure 1).

Beyond antibiotics, non-steroidal anti-inflammatory drugs, hormones, and antiepileptic drugs are also widely present in the environment [11,12,13]. Spanish studies have ranked the environmental indices of pharmaceuticals and personal care products [14], while in surface water and drinking water in southern Brazil various pesticides and PPCPs have been detected [15]. These pharmaceuticals, even at low concentrations, may produce chronic toxic effects on aquatic organisms, disrupt endocrine systems, affect reproduction and development, alter community structure, and thereby threaten ecosystem functions [16,17] (Table 1).

### 1.2. Applicability Boundaries and Limitations of Conventional Treatment Technologies

Conventional water treatment technologies exhibit clear applicability boundaries when facing the challenge of pharmaceutical pollution. Although physicochemical treatment methods are significantly effective in removing conventional pollutants, they encounter multiple dilemmas when dealing with pharmaceutical pollutants [20] (Table 2).

Coagulation–flocculation is relatively low-cost and effective for removing suspended solids and colloids; however, it has limited efficiency for dissolved pharmaceutical molecules and generates substantial chemical sludge that requires further disposal [1]. Adsorption technology (such as activated carbon adsorption) shows good removal effects for various pharmaceuticals, but adsorbent regeneration is costly, requiring frequent replacement, and only achieves phase transfer of pollutants rather than true degradation [21,22]. Membrane separation technologies (ultrafiltration, nanofiltration, reverse osmosis) can achieve efficient separation, but membrane fouling is prominent, energy consumption is high, and concentrate disposal remains an unresolved challenge [23]. Electrocoagulation has wide applicability but its energy-intensive nature limits large-scale application [24,25].

Advanced oxidation technologies (including ozonation, photocatalysis, electrochemical oxidation, etc.) have been highly anticipated for their ability to generate reactive oxygen species to attack organic molecules [26]. These technologies can achieve effective removal of recalcitrant organic pollutants but often require complex equipment, high operational costs, and may generate transformation products with unknown toxicity [26,28]. Studies show that although photocatalysis can achieve complete mineralization of organic pollutants, issues such as catalyst recovery, scale-up, and adaptability to environmental conditions remain to be resolved [27,29].

Notably, the removal efficiency of pharmaceuticals in conventional wastewater treatment plants varies considerably [31]. Although five wastewater treatment plants in California achieved over 90% removal for 14 pharmaceuticals, triclosan and octylphenol still existed in sludge at average concentrations of 1505 ng/g and 1179 ng/g, respectively. A survey of three wastewater treatment plants in Xiamen, China, revealed that 22 out of 48 target pharmaceuticals were detected in over half of the effluent samples, with ofloxacin reaching an average concentration of 2300 μg/kg in sludge. More concerning is that pharmaceutical pollutants have even been detected in drinking water [32]—concentrations in eight tap water samples ranged from not detected to 39 ng/L, and in eleven mineral water samples from 1 to 40 ng/L; Testing of 40 brands of bottled water in France (representing 70% of the market share) for 330 compounds detected no pharmaceutical ingredients [33]. This phenomenon indicates that existing drinking water treatment processes also have gaps in pharmaceutical removal capacity.

### 1.3. Limitations of Single Biotechnology: Respective Dilemmas of Enzymatic Catalysis and Microbial Degradation

Biological treatment technologies have attracted attention for their environmental friendliness and sustainable potential, but both free enzyme catalysis and microbial degradation face inherent limitations when applied alone.

The advantages of free enzyme technology lie in high catalytic efficiency, strong substrate specificity, and mild reaction conditions [34,35]. However, poor enzyme stability is its critical weakness—free enzymes are sensitive to environmental conditions (pH, temperature, ionic strength) and are easily inactivated (Table 3).

They are susceptible to protease attack or adsorption loss in complex environmental matrices; and they are difficult to recover and reuse, leading to high treatment costs [39,40]. Research by Sun Jian’s team at South China Agricultural University indicates that individual degrading enzymes face practical application challenges including high cost and poor stability, limiting their large-scale application [41,42] (Table 4).

Microbial degradation technology exhibits different advantages and limitations. Microorganisms possess complete metabolic networks, enabling deep mineralization of pollutants, and can self-propagate for sustained functionality [47,48]. However, microorganisms grow slowly, are sensitive to environmental conditions, and treatment efficiency is constrained by factors such as substrate concentration, co-metabolic substrates, and community structure. More critically, microorganisms have selective substrate spectra with limited degradation capacity for certain recalcitrant pharmaceutical pollutants; in composite pollution systems, interactions between different pollutants may inhibit microbial activity; and high concentrations of pharmaceuticals can be toxic and inhibitory to microorganisms [49]. Studies show that environmental stresses such as osmotic stress significantly affect the growth and metabolism of degrading bacteria, hindering their remediation function in polluted environments [50].

### 1.4. Synergistic Integration: The Inevitable Direction to Break Through Bottlenecks

The limitations of single technologies have given rise to the concept of “synergistic integration”. The high efficiency of enzymes and the complete metabolic networks of microorganisms are naturally complementary—enzymes can rapidly initiate reactions and break through recalcitrant molecular structures, while microorganisms can utilize their metabolic diversity to achieve deep mineralization; microorganisms can provide stable microenvironments for enzymes, while enzymes can relieve toxicity inhibition for microorganisms [51,52]. This “complementary and mutually reinforcing” characteristic makes enzyme–microbe synergistic platforms the inevitable choice to break through current bottlenecks.

**Figure 2:**
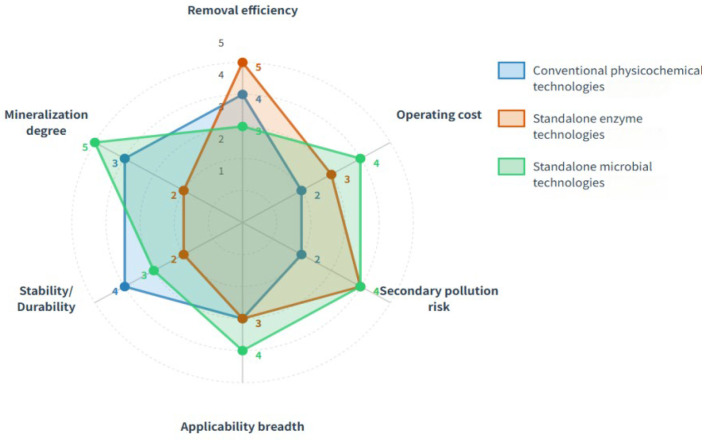
Comparison of limitations between conventional treatment technologies and single biotechnology.

Recent research progress provides strong support for this direction. The microbial consortium-based compound enzyme (MCE) demonstrates superior performance in food waste hydrolysis and antibiotic resistance gene removal compared to commercial enzymes and microbial monomer compound enzymes [53]. Interspecies synergistic interactions mediated by cofactor exchange induce biofilm formation, enhancing the environmental stress tolerance of microbial communities [50]. Co-immobilization technology integrates enzymes and cells on the same carrier, achieving efficient enhancement of cascade catalytic processes [54]. These advances collectively indicate that the synergistic integration of enzymes and microorganisms is opening new pathways for pharmaceutical pollution control (Figure 2).

## 2. Deconstruction of Synergistic Mechanisms: Complementarity and Enhancement of Enzyme and Microbial Platforms

**Figure 3:**
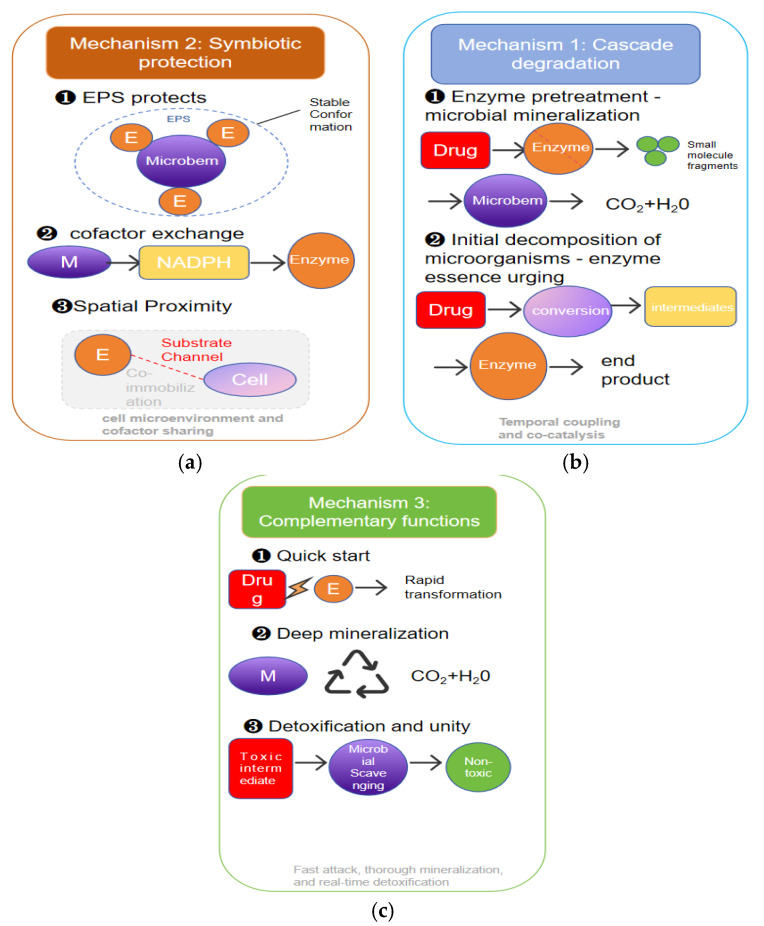
Schematic diagram of the three core mechanisms of enzyme–microbe synergy. (**a**) Enzyme pretreatment–microbial mineralization is a typical forward cascade. It can cleave large molecules or recalcitrant structures into small fragments, creating conditions for subsequent microbial degradation. “Microbial initial degradation–enzyme precision catalysis” is a backward cascade: after microorganisms convert drug molecules into specific intermediate structures, highly selective enzymes subsequently intervene to drive the transformation toward completion. (**b**) The polysaccharides, proteins, nucleic acids, and lipids in EPS can interact with enzyme molecules, providing a stable microenvironment for them. Microbial metabolic activities can produce cofactors required for enzymatic catalysis, compensating for the deficiency of free enzyme systems that require exogenous addition. The close spatial proximity between enzymes and microorganisms can significantly enhance reaction efficiency. (**c**) Enzymes can achieve rapid transformation of pharmaceutical pollutants within a short period of time. By virtue of their complete intracellular metabolic networks, microorganisms can continuously decompose intermediate metabolites until they are ultimately converted into CO_2_ and H_2_O. The presence of microorganisms facilitates the concurrent detoxification of the potentially toxic byproducts generated from enzymatic reactions.

The synergistic effect between enzymes and microorganisms is not simply additive but achieves emergent properties of “1 + 1 > 2” based on multiple mechanisms. Understanding these intrinsic mechanisms forms the theoretical foundation for rationally designing and optimizing synergistic platforms (Figure 3).

### 2.1. Cascade Degradation: Temporal Coupling of Synergistic Catalysis

Cascade degradation is the most intuitive mechanism of enzyme–microbe synergy. In this mode, enzymes and microorganisms respectively undertake different stages of the degradation process, forming functional temporal coupling [55].

Enzyme pretreatment–microbial mineralization is a typical forward cascade: many pharmaceutical molecules have complex structures and low bioavailability, making them difficult for microorganisms to directly uptake and metabolize. Enzymes, as “pioneer catalysts,” can cleave large molecules or recalcitrant structures into small fragments, increasing their water solubility and bioavailability, creating conditions for subsequent microbial degradation. Research by Zhonghu Bai’s team at Jiangnan University demonstrated a similar cascade strategy—constructing an efficient dual-enzyme cascade catalytic system for PET degradation by displaying PETase mutants and MHETase on the bacterial surface, achieving a degradation rate 51 times higher than free enzymes [56]. This strategy provides a methodological reference for pharmaceutical pollutant treatment: for complex pharmaceutical molecules, multi-enzyme cascade systems can be designed for initial breakdown, followed by complete mineralization through microbial networks.

In addition to forward cascade, there exists a reverse pathway that can be summarized as the “microbial initial degradation–enzyme precision catalysis” mode. In this process, microorganisms take the lead: either through their secreted extracellular enzymes or relying on their own metabolic pathways, they convert pharmaceutical molecules into specific intermediate structures; once this step is completed, highly selective enzymes intervene to catalyze key reaction steps, driving the transformation to completion. This division of labor strategy is particularly critical in dealing with composite pollution systems—the breadth of substrate spectrum in microbial communities enables them to handle multiple components, while the precise catalysis of key enzymes ensures targeted removal of recalcitrant substrates [53].

Further metabolic pathway analysis reveals that pretreatment with microbial consortium-based compound enzymes significantly enhances the catalytic efficiency of carbohydrate-active enzymes. Specifically, the abundance of genes involved in cellulose and starch degradation, polysaccharide synthesis, ABC transporters, and global regulation-related processes shows an increasing trend; conversely, genes related to paired formation systems, two-component regulatory systems, and quorum sensing show decreased abundance. This gene expression pattern, with some increasing and others decreasing, on one hand strengthens the hydrolysis process, and on the other hand effectively inhibits the spread of antibiotic resistance genes [53].

### 2.2. Symbiotic Protection: Contribution of Microbial Microenvironments to Enzyme Stability

The instability of enzymes is a major obstacle to their practical application, but the presence of microorganisms provides natural protective environments for them. This symbiotic protection mechanism manifests at multiple levels [57].

Protective effect of extracellular polymeric substances. Extracellular polymeric substances (EPSs) secreted by microorganisms constitute the matrix skeleton of biofilms and also provide stable microenvironments for extracellular enzymes. The polysaccharides, proteins, nucleic acids, and lipids in EPS can interact with enzyme molecules, restricting conformational fluctuations, preventing denaturation and aggregation, and enhancing tolerance to temperature, pH, proteases, and other factors [57]. Research reveals that under micro/nanoplastic stress, the responses of EPSs in activated sludge are governed by reactive oxygen species-mediated regulatory networks [58]. Micro/nanoplastics can directly bind with key antioxidant enzymes such as superoxide dismutase and catalase (binding energy < −5 kcal/mol), inhibiting their enzyme activity and reducing related gene abundance, leading to intracellular ROS accumulation, which in turn drives microbial community succession towards EPS-producing bacteria, strengthening EPS secretion to cope with stress [58]. This finding indirectly confirms the critical role of EPSs in protecting extracellular enzyme activity.

The synergistic effect of cofactor exchange: Microbial metabolic activities can produce cofactors required for enzymatic catalysis, compensating for the deficiency of free enzyme systems that require exogenous addition, significantly reducing costs [59]. The South China Agricultural University team constructed a multi-enzyme complex FerTiG mimicking microcompartment structures, integrating the degradation module Tet(X4) and the recycling module GDH—GDH catalyzes glucose oxidation to provide NADPH required by Tet(X4), reducing costs by 10 times while improving reaction efficiency by approximately seven times [41]. This “cofactor cycling” model precisely mimics the natural mechanism of cofactor exchange in microbial communities [50].

Further research indicates that interspecies cofactor exchange can enhance the environmental stress tolerance of microbial communities. In a synergistic consortium constructed with *Rhodococcus ruber* and *Epilithonimonas zeae*, multi-omics analysis and genome-scale metabolic model simulations revealed that the vitamin B12-dependent methionine–folate cycle is a key pathway enhancing hyperosmotic stress tolerance. The consortium promotes biofilm formation by exchanging S-adenosylmethionine and riboflavin (a cofactor required for vitamin B12 biosynthesis), thereby improving overall stress tolerance [50].

The spatial proximity effect: Close spatial proximity between enzymes and microorganisms can significantly improve reaction efficiency. Co-immobilization technology confines enzymes and cells to the same microenvironment, shortening substrate and product diffusion distances, allowing intermediate products to be rapidly utilized by adjacent cells, avoiding loss or accumulation inhibition [60,61]. Nankai University’s team created a covalent organic framework co-immobilization platform, integrating inulinase and *E. coli* within COF armor, achieving efficient cascade catalysis and maintaining >90% of initial catalytic efficiency after 7 days of continuous reaction [54].

### 2.3. Functional Complementarity: Unification of Rapid Initiation and Deep Mineralization

The functional complementarity between enzymatic catalysis and microbial metabolism achieves the unification of “speed” and “depth” in the treatment process [55].

Rapid initiation capability of enzymes: The catalytic efficiency of enzymes far exceeds microbial metabolic rates, enabling rapid transformation of pollutants in a short time. This is of great significance for responding to sudden pollution events or treating high-concentration wastewater [62,63]. Additionally, enzymes can act on targets inaccessible to microorganisms, such as cell membrane-impermeable substrates. Jiangnan University’s research team pointed out that since PET can hardly cross cell membranes to reach intracellular space, using microbial degradation alone is ineffective, while displaying PETase and MHETase on the cell surface constructs an efficient dual-enzyme cascade catalyst [56].

Deep mineralization capability of microorganisms: Enzymatic reactions typically stop converting pollutants into specific intermediate products, may not achieve complete degradation, and some transformation products may even retain ecological risks [64]. In contrast, microorganisms, with their complete intracellular metabolic networks, possess the ability to continuously decompose these intermediate products until ultimately converting them into CO_2_ and H_2_O, thereby achieving true detoxification of pollutants [65]. This unique ability to completely mineralize organic substances is precisely what single-enzyme systems find difficult to achieve.

The unification of detoxification and removal: More notably, the presence of microorganisms can simultaneously eliminate the potential toxicity of enzymatic reaction products. Studies indicate that some enzymatic reaction intermediates may have toxicity or biological activity exceeding that of parent compounds, and if not promptly eliminated, could cause secondary pollution. Timely microbial intervention can precisely cut this risk pathway, achieving the dual goals of pollutant removal and toxicity reduction [66]. Certain enzymes can also directly act on toxic compounds, reducing the stress imposed on microorganisms and thereby protecting their metabolic activity. Ochratoxin A (OTA) is a mycotoxin known for its strong nephrotoxicity and carcinogenicity. Research has revealed that the strain *Lysobacter* sp. CW239 harbors a highly active amidohydrolase, ADH2, and a carboxypeptidase, CP4, exhibiting low activity. The former efficiently cleaves the critical toxic groups of OTA, while the latter facilitates the adequate expression of the primary enzyme through regulatory functions. The synergy between these two enzymes significantly promotes the microbial degradation and biotransformation of OTA [67].

## 3. Construction of Synergistic Platforms: From Enhancement Strategies to Engineering Applications

**Figure 4:**
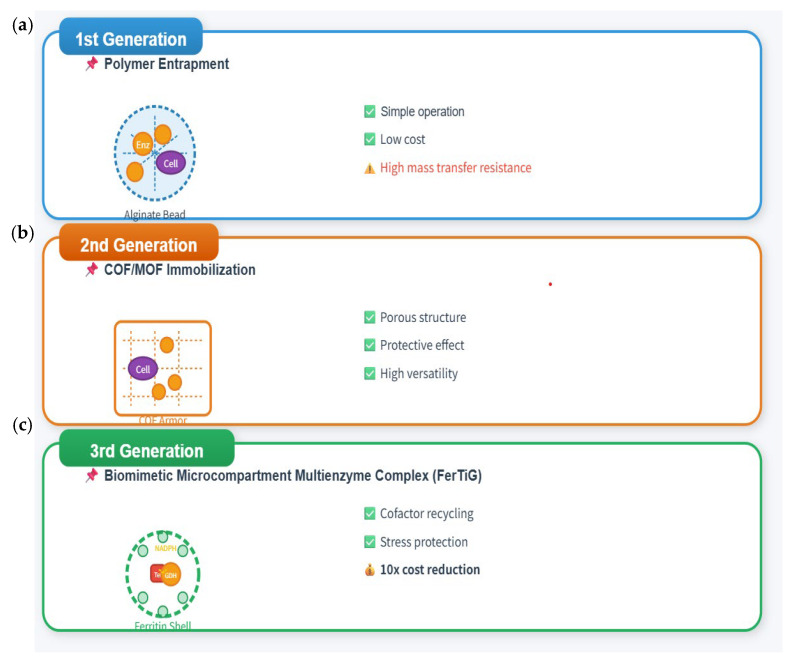
Construction strategies of engineered biofilm and synthetic biology-engineered bacteria. (**a**) Enzymes and cells are co-dispersed in natural or synthetic polymers, and subsequently converted into immobilized particles via crosslinking or gelation [68]. (**b**) A uniform covalent organic framework (COF) armor is uniformly coated onto the cell surface, and enzymes are subsequently immobilized within this armor, enabling efficient co-localization of the enzymes and cells [54]. (**c**) The multi-enzyme complex FerTiG integrates the degradation module Tet(X4), the cofactor recycling module GDH, and the protective module Ferritin. GDH catalyzes the oxidation of glucose to provide the NADPH required by Tet(X4). Meanwhile, Ferritin forms a dense compartment around the two functional enzymes, thereby protecting them from adverse environmental factors such as high temperature, low pH, high osmolarity, and ultraviolet irradiation [41].

In-depth understanding of synergistic mechanisms provides theoretical guidance for the design and construction of synergistic platforms. Researchers have developed multiple technological pathways to achieve the effective integration of enzymes and microorganisms (Figure 4).

### 3.1. Co-Immobilization Technology: Construction of Artificial Synergistic Systems

Co-immobilization is an effective strategy to confine enzymes and microbial cells to the same carrier, achieving spatial proximity and synergistic catalysis. Based on different immobilization carriers, it can be divided into multiple types.

Metal–organic framework immobilization (MOF) is another important direction. Metal–organic frameworks (MOFs) possess high specific surface area, tunable pore structure, and good stability, showing broad prospects in the field of enzyme immobilization [69].

### 3.2. Biofilm Platforms: Natural Synergistic Ecosystems

Biofilms represent the main form of microbial existence in nature and serve as natural platforms for enzyme–microbe synergy [70]. Within biofilms, microorganisms are embedded in self-secreted EPS matrices, and extracellular enzymes can be retained within the EPS network, forming highly organized functional units [71,72].

Application of natural biofilms: Researchers can directly utilize natural biofilms with degradation functions to treat pharmaceutical pollutants. The metabolic diversity of microorganisms in biofilms can address multiple pollutants, while EPS-retained extracellular enzymes contribute rapid degradation capabilities [71]. The activated sludge process essentially utilizes microbial aggregates (flocs, biofilms) to treat wastewater, and its removal of pharmaceuticals has been extensively studied.

Construction of engineered biofilms. The development of synthetic biology enables researchers to rationally design and modify biofilms [73]. A research team from Bilkent University in Turkey utilized *E. coli* biofilm protein CsgA as a scaffold, fusing it with two types of laccases through the SpyTag–SpyCatcher system to construct an engineered biofilm platform capable of degrading ciprofloxacin [74]. Mass spectrometry analysis and cell viability assays confirmed that the designed biofilm material successfully degraded fluoroquinolone antibiotics. The advantages of this strategy include: biofilms can exist stably for long periods, self-renew, adapt to flowing environments, and engineered modifications offer modularity and programmability.

Regulatory mechanisms of biofilm formation: Understanding the molecular mechanisms of biofilm formation helps optimize the design of synergistic platforms. Research shows that interspecies cofactor exchange can induce biofilm formation, enhancing the environmental stress tolerance of microbial communities [50]. In the synergistic consortium of *Rhodococcus ruber* and *Epilithonimonas zeae*, the vitamin B12-dependent methionine–folate cycle was identified as a key pathway enhancing hyperosmotic stress tolerance, with the consortium promoting biofilm formation by exchanging S-adenosylmethionine and riboflavin. This finding provides a new strategy for constructing efficient and stable synergistic platforms—by regulating cofactor supply, biofilm formation and function can be directionally enhanced.

### 3.3. Synthetic Biology-Engineered Bacteria: From Single Cells to Multifunctional Platforms

**Figure 5:**
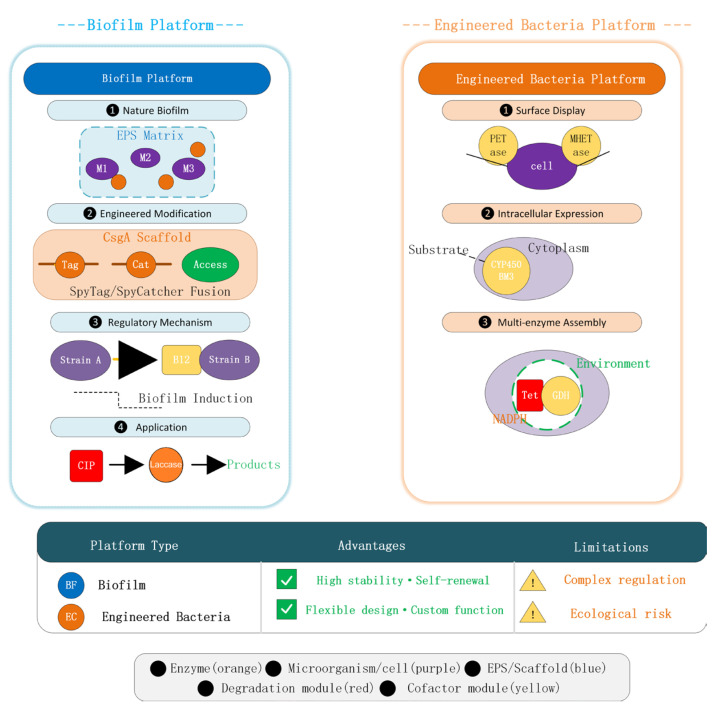
Construction strategies for engineered biofilms and synthetic biology-engineered bacteria.

Synthetic biology enables researchers to transcend natural evolutionary limitations and construct engineered strains with customized functions [75]. Within the enzyme–microbe synergistic framework, engineered bacteria applications present multiple modes. As shown in Figure 5 below.

Construction of surface display systems: Displaying enzymes on cell surfaces avoids substrate transmembrane transport limitations while achieving co-localization of enzymes and cells [76]. Jiangnan University’s team constructed an efficient dual-enzyme cascade catalyst by displaying PETase mutants and MHETase on the surface of *E. coli* and Pseudomonas putida using autotransporter proteins [56]. By modifying host cells, co-expressing molecular chaperones, and evolving the autotransporter YeeJ, the surface display efficiency of rate-limiting enzymes was significantly enhanced, increasing the PET degradation rate to 3.85 mM/d, 51 times higher than free enzymes. Cell catalyst EC9F retained 38% and 30% of initial activity after 22 cycles of BHET degradation and 3 cycles of PET degradation, respectively.

Application of intracellular expression systems: For membrane-permeable substrates, intracellular expression of engineered enzymes is equally effective [77]. A research team from NOVA University Lisbon expressed the CYP102A1 mutant enzyme (BM3 MT35) in Bacillus megaterium and Chlamydomonas reinhardtii respectively for degradation of the herbicide diuron [78]. Transgenic *B. megaterium* degraded 65% of diuron after 5 days in TB medium, and 45% and 15% in synthetic wastewater and municipal wastewater, respectively; transgenic *C. reinhardtii* expressing P450 BM3 MT35 in chloroplasts showed significantly higher diuron degradation (52%) compared to the wild type (6%).

Intracellular assembly of multi-enzyme complexes: Mimicking bacterial microcompartment structures to assemble multi-enzyme complexes intracellularly can further improve catalytic efficiency [79]. Although FerTiG constructed by Sun Jian’s team was assembled in vitro, its design concept can be extended to intracellular systems—through protein scaffolds or compartmentalization signals, multi-enzyme systems can be localized to specific cellular regions to achieve efficient cascade reactions (Table 5).

### 3.4. Treatment Efficacy for Typical Pharmaceutical Pollutants

**Figure 6:**
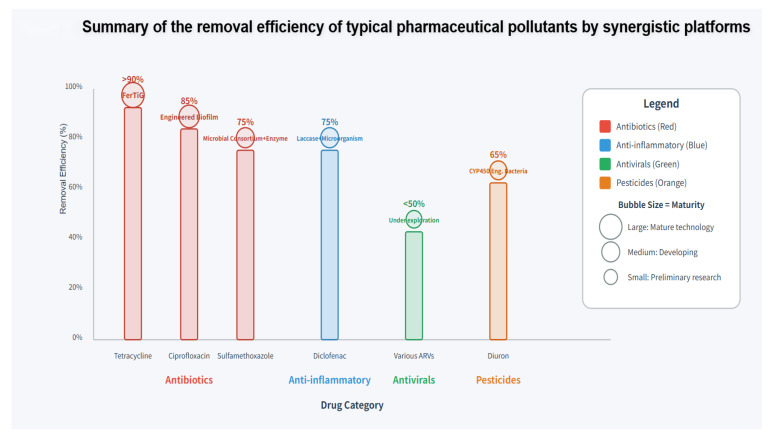
Summary of removal efficacy of synergistic platforms for typical pharmaceutical pollutants.

Synergistic platforms have achieved significant progress in treating various types of pharmaceutical pollutants (Figure 6).

Antibiotics are the most intensively studied drug category [80], and tetracycline antibiotic degradation has been the most extensively studied, with FerTiG multi-enzyme complexes efficiently decomposing tetracycline residues driven by glucose [41]. For fluoroquinolone antibiotics, laccase-type enzymes have been confirmed to attack synthetic antibiotics such as ciprofloxacin, with engineered biofilm platforms successfully achieving their degradation [74]. Sulfonamide antibiotics are widely present in the environment, with sulfamethoxazole frequently detected in water bodies in East Africa [6].

Anti-inflammatories and hormones have also received attention. Non-steroidal anti-inflammatory drugs such as diclofenac and ibuprofen are commonly detected in wastewater treatment plant effluents [81], with limited efficiency when treated with single biological systems. Synergistic platforms are expected to overcome this bottleneck. Laccase degradation of anti-inflammatories has been studied [82].

Antiviral drugs research is relatively limited but increasing in importance [83]. The presence of antiretroviral drugs in the environment in East Africa has been confirmed, and removal needs are gradually receiving attention [84].

Pesticides, although not typical drugs, have similar structures and properties, allowing methodological cross-reference [85]. CYP102A1 mutant enzymes expressed in transgenic microorganisms have been successfully used for efficient diuron degradation [78] (Table 6).

## 4. Towards Green Pharmaceuticals: Closed-Loop Value and Future Prospects of Synergistic Governance

### 4.1. Paradigm Shift from End-of-Pipe Treatment to Full-Cycle Management

In the past, the focus of pharmaceutical pollution control has always been on the “end-of-pipe”—the treatment stage before wastewater enters the natural environment [86]. However, with the deepening of green chemistry concepts and the gradual rise of circular economy models, this long-standing traditional paradigm is undergoing profound transformation [87,88]. To fundamentally solve the pharmaceutical pollution dilemma, it is urgent to establish a management perspective covering the full lifecycle: not only moving the control point forward to the drug design and production stages, but also extending it backward to consumption and final disposal [89,90].

Looking back at this transformation context, the emergence and implementation of the “ecopharmacovigilance” concept is undoubtedly the most iconic aspect [91]. This concept directly addresses the environmental impact of drugs throughout the entire process from research and development, production and use to final disposal, advocating green design, rational use, and standardized disposal as starting points to curb the channels of drug input into the environment at the source [92]. This is particularly evident in relevant research in East Africa—scholars call for the promotion of ecopharmacovigilance implementation within the “One Health” framework, attempting to resolve the intertwined dilemma of pharmaceutical pollution and antibiotic resistance spread through multiple means, including strengthening environmental monitoring, improving regulatory enforcement, upgrading sewage treatment capacity, and promoting green pharmaceutical technologies [93]. Meanwhile, international institutions such as the EU and OECD have successively issued strategic documents and policy guidance, providing systematic institutional responses to the increasingly prominent pharmaceutical issues in the environment [94,95].

The green pharmaceutical concept emphasizes the incorporation of environmental considerations into drug research, development, and production processes. This includes: designing easily biodegradable drug molecules, optimizing synthesis routes to reduce waste generation, adopting green processes such as continuous flow manufacturing, and developing environmentally friendly formulations [96]. These efforts complement end-of-pipe treatment, jointly constructing a more sustainable pharmaceutical lifecycle.

### 4.2. Closed-Loop Value of Synergistic Platforms: Resource Recovery and Process Integration

Beyond end-of-pipe treatment, enzyme–microbe synergistic platforms exhibit broader closed-loop value [97].

Resource utilization of degradation products: Although complete mineralization of pharmaceutical molecules to CO_2_ and H_2_O is the ideal goal, converting degradation products into recoverable resources is potentially more sustainable. For example, ammonium released during nitrogen-containing drug degradation can be recovered as fertilizer; small molecule organic acids generated from aromatic ring-containing drug degradation can provide carbon sources for microorganisms [98]. Although this direction is still in early exploration, its application prospects are worth anticipating.

The reverse integration of treatment systems with pharmaceutical processes: Embedding synergistic treatment systems into pharmaceutical production processes can achieve “treatment while producing”. Continuous flow enzyme–cell immobilized reactors can be directly linked with production lines for online treatment of process wastewater containing drug residues, thereby reducing discharge loads. The continuous-flow device developed by Nankai University’s team provides a feasible proof-of-concept prototype for this technological pathway [54].

Application of green solvents and auxiliaries: The construction of synergistic platforms can also practice green chemistry principles [99]. Specific measures include: using renewable biomass materials as immobilization carriers, utilizing cofactors produced by microbial metabolism to replace exogenous additions, and introducing biosurfactants and other biological auxiliaries to enhance catalytic efficiency [100].

### 4.3. Challenges and Constraints: From Laboratory to Practical Application

Enzyme–microbe synergistic platform technologies have successfully transitioned from the laboratory to both pilot-scale and industrial applications. At the pilot scale, 50–300 L fermentation systems have been validated for process optimization and scale-up trials. At the industrial scale, production capacities at the 20, 50, and even hundred-ton levels have been achieved, with certain processes now running reliably on ten-thousand-ton production lines. Moreover, these technologies have been deployed in over 100 products and support nearly 300 enterprises, reaching Technology Readiness Levels (TRLs) of 7 to 9. Nevertheless, transitioning from laboratory research to practical engineering applications still faces multiple challenges [101].

Stability issues in complex matrices: The composition of actual wastewater is far from simple—various organic substances, inorganic salts, and suspended solids coexist, and this highly complex matrix environment may interfere with enzyme activity, affect microbial metabolic homeostasis, and even threaten the structural integrity of immobilization carriers [58]. In real wastewater matrices, enzyme stability typically decreases by 50–70%, while the mechanical strength of immobilized carriers declines by approximately 50% over a 30-day period. Notably, EPS protection improves thermal stability by approximately 4.5-fold, suggesting that the symbiotic protection mechanism possesses considerable engineering relevance.

The cost-effectiveness of scale-up: Regarding cost-effectiveness, advanced technologies such as co-immobilization and synthetic biology modification currently still face practical constraints of relatively high preparation costs, and their marginal costs and economic benefits in scaled production require further examination [102]. Although the enzyme–microbe synergistic platform incurs substantially higher treatment costs at the laboratory scale (15–30 USD/m^3^) compared to conventional physicochemical methods (5–10 USD/m^3^), these costs are projected to decrease to 3–8 USD/m^3^ upon industrial scale-up, corresponding to a 70–80% reduction. Notably, the material costs of advanced carriers like COFs and MOFs are expected to decline by 90% with large-scale manufacturing, positioning this as a pivotal avenue for achieving economic feasibility.

Systematic assessment of ecological safety: Once engineered bacteria are released into the natural environment, the potential ecological risks cannot be ignored—including the possibility of horizontal gene transfer, the risk of disrupting indigenous population balance, and even effects on non-target organisms [103]. When live engineered bacteria are released into the environment, the resulting gene transfer frequency ranges from 10^−5^ to 10^−3^ [104], and the bacteria can survive for 7 to 28 days. Moreover, their presence induces non-target effects, including a 15–25% decrease in the diversity of indigenous microbial communities and a 20–40% suppression of microfauna reproduction. In marked contrast, cell-free systems such as FerTiG display an approximately 100- to 1000-fold-lower ecological risk, thus offering a substantially safer alternative in terms of biosafety.

Even after enzymatic pretreatment followed by microbial mineralization, pharmaceutical pollutants may still undergo incomplete degradation or remain inhibitory. This phenomenon is well illustrated in studies on itaconic acid (IA) production from lignocellulosic biomass, where it was found that even after enzymatic saccharification to release fermentable sugars, the hydrolysate still contained various inhibitory compounds that significantly hampered subsequent fermentation [105]. For instance, sugar degradation products such as furfural and 5-hydroxymethylfurfural (HMF), acetic acid generated from hemicellulose degradation, and phenolic compounds derived from lignin degradation can all act as microbial growth inhibitors.

Lag in regulatory frameworks: There is a significant time lag between the pace of iteration of emerging technologies and the update pace of existing regulatory systems [106]. With the annual growth rate of technological advancement ranging from 15% to 20%, which substantially surpasses the frequency of regulatory framework revisions (once every 5 to 10 years), a technology–policy time lag of 4 to 7 years has emerged. Notably, the regulatory categorization of nascent platforms—including cell-free systems such as FerTiG and engineered biofilms—remains unresolved, posing a major bottleneck to their real-world deployment.

### 4.4. Future Research Directions: Intelligent Regulation and Multi-Omics Guidance

Looking forward, research on enzyme–microbe synergistic platforms can be expanded in the following directions.

Construction of intelligent regulation systems: Introducing sensor–actuator circuits into synergistic platforms achieves real-time response and adaptive regulation to environmental changes [107], for example constructing promoters that sense pollutant concentrations to regulate enzyme expression levels, or utilizing quorum-sensing systems to coordinate the division of labor in microbial communities at different stages [108].

Multi-omics guided optimization of synergistic systems: Integrating multi-omics technologies including genomics, transcriptomics, proteomics, and metabolomics, combined with genome-scale metabolic models, can systematically analyze interaction mechanisms among microbial communities, providing guidance for the rational design of synergistic systems [109]. Through metabolic model simulation, optimal strain ratios, substrate supply strategies, and environmental condition parameters can be predicted, significantly shortening optimization cycles [110].

Exploitation of non-model microbial resources. Current research mostly focuses on model strains such as *E. coli* and yeast, while non-model microorganisms widely present in the environment harbor rich degradation potential and adaptation mechanisms. Isolating efficient degrading consortia from polluted environments, analyzing their synergistic mechanisms, and transplanting their functional elements into engineered strains are directions worth exploring [48,111].

AI-assisted enzyme engineering: The application of machine learning in enzyme design and optimization is becoming increasingly widespread. For example, protein language models (pLMs) have emerged as a mainstream framework for enzyme function prediction and design [112]. These models are pre-trained directly on large-scale protein sequence datasets to learn the intrinsic mapping between sequence and function. As a result, they can predict beneficial mutations without requiring explicit three-dimensional structural information, thereby significantly reducing dependence on structural biology. The discovery and modification of novel degrading enzymes can also be accelerated, thereby enhancing the overall treatment efficacy of synergistic platforms.

Innovative paradigm of interdisciplinary integration: Research on enzyme–microbe synergistic platforms is at the intersection of chemistry, biology, materials science, environmental engineering, and synthetic biology [113]. Deep interdisciplinary collaboration is expected to catalyze breakthroughs, promoting the advancement of pharmaceutical pollution control towards a greener and more sustainable future [114].

## 5. Conclusions

As a class of persistently emerging trace pollutants, pharmaceutical contaminants have raised widespread concern regarding their potential risks to ecosystems and public health. Conventional physicochemical treatment technologies face common challenges when dealing with such low-concentration, highly toxic, and structurally complex pollutants, including high costs, significant secondary pollution risks, and incomplete degradation. Standalone enzymatic catalysis and microbial degradation pathways each have their own limitations, making it difficult to achieve a balance among efficiency, stability, and economic feasibility. Against this backdrop, the synergistic integration platform of enzymes and microorganisms, leveraging its three core mechanisms—cascade degradation, symbiotic protection, and functional complementarity—has demonstrated unique potential for achieving efficient, thorough, and sustainable pharmaceutical pollutant remediation.

This review systematically summarizes research progress in this field, with particular emphasis on the current status and trends of synergistic construction strategies, including co-immobilization technology, engineered biofilms, and synthetically engineered bacteria. Notably, current research has enabled this platform to achieve significant efficacy in the treatment of representative pharmaceutical pollutants such as tetracycline, ciprofloxacin, and sulfamethoxazole, with some systems exhibiting degradation efficiencies and environmental compatibility superior to conventional physicochemical processes. Nevertheless, the translation of this platform from laboratory research to practical engineering applications still faces multiple real-world constraints, including insufficient long-term operational stability in complex wastewater matrices, high cost bottlenecks associated with scale-up production, potential ecological safety risks of genetically engineered strains, and a marked lag in regulatory frameworks in the era of synthetic biology.

From a broader academic perspective, the value of the enzyme–microbe synergistic platform should not be confined to the level of “end-of-pipe” treatment, but rather re-examined within the larger context of “lifecycle green pharmaceuticals”. Promoting the paradigm shift from passive end-of-pipe treatment to proactive source prevention and process integration requires the establishment of a circular economy loop encompassing “design–production–use–disposal–regeneration.” This transformation depends not only on the continuous improvement of catalytic components (enzymes and strains), but also calls for deep interdisciplinary convergence—including the fine reprogramming of metabolic networks through synthetic biology, intelligent design and optimization of enzyme structure and function via artificial intelligence, and the development of high-performance immobilization carriers through materials science. Furthermore, a truly “green pharmaceutical lifecycle” necessitates collaborative actions among policymakers, pharmaceutical companies, and research institutions to integrate sustainability metrics into the full-cycle assessment framework of drug research, development, and production.

Looking forward, empowered by multi-omics data-driven approaches, artificial intelligence-assisted design, and automated closed-loop evolution platforms, the enzyme–microbe synergistic platform is expected to overcome current bottlenecks in stability and cost-effectiveness, thereby accelerating the integrated convergence of remediation technologies and pharmaceutical processes. Only by unblocking the entire chain from laboratory discovery through pilot validation to engineering scale-up, while simultaneously improving risk assessment and management frameworks for synthetic biology products, can this platform unlock its full transformative potential in environmental remediation and green chemical engineering, ultimately moving toward an ecologically safe and economically viable sustainable management paradigm.

## Figures and Tables

**Table 1:** Environmental concentrations, sources, and ecological risks of typical pharmaceutical pollutants.

Drug Category	Representative Drug	Environmental Matrix	Detected Concentration Range	Main Sources	Ecological Risk (RQ)	References
Antibiotics	Ciprofloxacin	Surface water	nd–14.3 μg/L	Aquaculture wastewater, domestic sewage	3.5–40.6	[4,6,7]
	Sulfamethoxazole	Surface water	nd–2.8 μg/L	Domestic sewage, medical wastewater	0.1–3.53	[4,6,16]
	Tetracycline	Sludge	89–2300 μg/kg	Aquaculture wastewater	-	[10,16]
	Ofloxacin	Sludge	2300 μg/kg (average)	Domestic sewage	-	[16]
Anti-inflammatory drugs	Diclofenac	Surface water	nd–1.2 μg/L	Domestic sewage	<0.1	[12,16]
	Ibuprofen	WWTP effluent	0.1–2.5 μg/L	Domestic sewage	<0.1	[12,13]
Hormones	Octylphenol	Sludge	1179 ng/g (average)	Industrial/domestic sewage	-	[12]
	Triclosan	Sludge	1505 ng/g (average)	Personal care products	-	[12]
Antiviral drugs	Various ARVs	Surface water	nd–3.2 μg/L	Medical wastewater	To be assessed	[6,18]
Pesticides	Diuron	Surface water	nd–0.8 μg/L	Agricultural runoff	0.1–0.5	[15,19]

nd: not determined.

**Table 2:** Comparison of advantages and disadvantages of conventional physicochemical treatment technologies for pharmaceutical pollutants.

Technology Type	Removal Mechanism	Advantages	Disadvantages	Example Drug Applications	References
Coagulation–flocculation	Charge neutralization, bridging adsorption	Simple operation, low cost, suitable for large scale	Low removal efficiency for dissolved drugs, large sludge production	Hydrophobic drugs	[1]
Adsorption	Physical/chemical adsorption	High removal rate, simple equipment	Phase transfer only (non-degradative), high adsorbent regeneration cost	Multiple drugs	[21,22]
Membrane separation	Sieving, charge repulsion	High separation efficiency, no chemical addition	Membrane fouling, high energy consumption, difficult concentrate disposal	Large molecule drugs	[23]
Electrocoagulation	In situ coagulant generation	Wide applicability, no external chemicals required	High energy consumption, electrode consumption	Antibiotics	[24,25]
Ozonation	Direct oxidation/·OH oxidation	Rapid reaction, no sludge production	Potential generation of toxic byproducts, complex equipment	Drugs with unsaturated structures	[26]
Photocatalysis	·OH oxidation	Complete mineralization possible, utilizes solar energy	Difficult catalyst recovery, limited scalability	Multiple drugs	[27,28,29]
Electrochemical oxidation	Direct/indirect oxidation	Strong oxidation capacity, good controllability	High energy consumption, limited electrode life	Refractory drugs	[26,30]
Fenton oxidation	·OH oxidation	Rapid reaction, simple equipment	Narrow pH range applicability, iron sludge generation	Multiple drugs	[26,30]

**Table 3:** Summary of inactivation mechanisms of enzymes induced by environmental factors.

Environmental Factor	Primary Inactivation Mechanism	Molecular-Level Explanation	Representative Data/Case	Reference(s)
pH deviation from optimum	Alteration of active site ionization state; induction of non-native association/aggregation	Change in protonation state of catalytic residues; surface charge alterations leading to aggregation	EstGtA2 forms a non-native associated state (300 nm apparent particle size) resistant to unfolding up to 95 °C at specific pH	[36]
Elevated temperature	Enhanced thermal vibration; conformational collapse; activity/stability trade-off	Disruption of hydrogen bonds and hydrophobic interactions; competition between kinetic acceleration and inactivation	Laccase tends to inactivate above 45 °C	[37]
High ionic strength	Charge screening promoting aggregation; direct inhibition of active sites	“Salting-out” effect disrupting the hydration layer; interference with metal cofactors by metal ions/halides	Fe(III) and Cu(II) significantly inhibit BPA conversion	[37]
Combined stress (pH + temperature)	Synergistic acceleration of inactivation	Inactivation follows first-order kinetics (kd); faster inactivation as conditions deviate from optimum	Serine protease exhibits minimal kd at pH 9 and 37 °C	[38]

**Table 4:** Comparison of free enzymes and immobilized enzymes: cost and stability data.

Parameter	Free Enzyme	Immobilized Enzyme	Improvement Factor	Reference(s)
Unit cost	~1 USD/cm^3^	~0.107 USD/cm^3^	~90% reduction	[43]
Residual activity after 6 h at 50 °C	40.63%	>85%	~2-fold increase	[44]
Residual activity after 6 h UV exposure	7.23%	92.88%	~13-fold increase	[44]
Optimal temperature	50 °C	55–60 °C	5–10 °C increase	[45]
Number of reusability cycles	1	9 cycles with ~50% retention	Multiple cycles	[46]
Storage stability	Days to weeks	>50% activity after 8 weeks	Significantly extended	[46]

**Table 5:** Main construction strategies and technical characteristics of enzyme–microbe synergistic platforms.

Synergy Strategy	Specific Technology	Carrier/Platform	Key Features	Advantages	Limitations
**Co-immobilization**	Polymer entrapment	Alginate, PVA	Physical entrapment	Simple operation, low cost	High mass transfer resistance
	COF immobilization	Covalent organic frameworks	Porous armor, co-localization	Enzyme protection, good substrate diffusion	Complex synthesis
	MOF immobilization	Metal–organic frameworks	High surface area, tunable pore size	High stability	Biocompatibility needs optimization
	Mimetic microcompartment composite	Ferritin shell	Multi-enzyme assembly, cofactor cycling	7× efficiency ↑, 10× cost ↓	Complex design
**Biofilm**	Natural biofilm	EPS matrix	Extracellular enzyme retention, community metabolism	High stability, self-renewal	Difficult regulation
	Engineered biofilm	CsgA scaffold	SpyTag/SpyCatcher fusion	Modular design, programmable	Long construction cycle
	Cofactor regulation	Biofilm	Induced formation by cofactor exchange	Enhanced stress tolerance	Complex mechanism
**Engineered bacteria**	Surface display	Bacterial surface	Enzymes displayed on cell surface	Overcomes substrate transmembrane limitation	Limited display efficiency
	Intracellular expression	Cytoplasm	Intracellular expression of engineered enzymes	Utilizes intracellular metabolism	Substrates require transmembrane transport
	Multi-enzyme intracellular assembly	Artificial microcompartment	Mimics bacterial microcompartments	Efficient cascade catalysis	Difficult assembly

Notes: ↑: increase; ↓: decrease.

**Table 6:** Summary of research cases on the treatment of typical pharmaceutical pollutants by synergistic platforms.

Drug Category	Specific Drug	Synergy Platform Type	Platform Composition	Experimental Conditions	Removal Efficiency	Key Findings	Cost Analysis
**Tetracyclines**	Tetracycline	Mimetic microcompartment multi-enzyme complex	FerTiG (Tet(X4) + GDH + Ferritin)	Glucose-driven, room temperature	>90% (24 h)	10× cost reduction, 7× efficiency improvement, strong stress resistance	10-fold cost reduction via cofactor recycling; glucose as low-cost driving fuel
**Fluoroquinolones**	Ciprofloxacin	Engineered biofilm	*E. coli* CsgA-laccase fusion	Flow system, room temperature	85% (48 h)	Long-term stable operation, modular design	Economical due to self-regenerating biofilm; no external enzyme supplementation required
**Sulfonamides**	Sulfamethoxazole	Microbial consortium + enzyme	Oriented microbial consortium-based compound enzyme	Wastewater treatment conditions	70–80%	Simultaneous ARGs removal	Cost-effective using agricultural/food residues (vine pruning, brewer’s spent grains) as enzyme production substrate; 50 U·L^−1^ low enzyme concentration
**Anti-inflammatory drugs**	Diclofenac	Laccase + microorganism	Free laccase + activated sludge	Batch experiment	75%	Laccase pretreatment enhances biodegradation	Moderate cost; reduces subsequent biotreatment burden
**Pesticides**	Diuron	Engineered bacteria (intracellular expression)	*B. megaterium* expressing CYP450 BM3	TB medium	65% (5 d)	45% in synthetic wastewater, 15% in municipal wastewater	Requires IPTG induction; cost of heterologous expression system
	Diuron	Engineered microalgae (chloroplast expression)	*C. reinhardtii* expressing CYP450 BM3	Light culture	52%	Wild type only 6%	Lower cost than bacterial system; light-driven, no inducer required
**Plastic monomers**	PET (methodological reference)	Surface displays dual enzymes	*E. coli* displaying PETase + MHETase	37 °C	3.85 mM/d	51× improvement over free enzyme, reusable	High reusability (3 cycles retain 30% activity); reduces long-term costs
**Multiple drugs**	14 drugs	WWTP (biofilm)	Activated sludge process	Actual WWTP	>90% (majority)	Triclosan and octylphenol still persisted in sludge	Low operational cost for existing infrastructure; sludge disposal adds expense

## Data Availability

Data sharing is not applicable to this article as no datasets were generated or analyzed during the current study.

## References

[1] Samal K., Mahapatra S., Hibzur Ali M. (2022). Pharmaceutical wastewater as Emerging Contaminants (EC): Treatment technologies, impact on environment and human health. Energy Nexus.

[2] Kotwani A., Joshi J., Kaloni D. (2021). Pharmaceutical effluent: A critical link in the interconnected ecosystem promoting antimicrobial resistance. Environ. Sci. Pollut. Res..

[3] Kimera Z.I., Mshana S.E., Rweyemamu M.M., Mboera L.E.G., Matee M.I.N. (2020). Antimicrobial use and resistance in food-producing animals and the environment: An African perspective. Antimicrob. Resist. Infect. Control.

[4] Tegegne A.A., Mekasha Y.T., Ayu A.A., Hasen G., Suleman S. (2024). A review on emerging pharmaceutical residues in Ethiopia: Occurrence, ecotoxicological aspects, and regulatory concerns. Front. Microbiol..

[5] Nantaba F., Wasswa J., Kylin H., Bouwman H., Palm W.U., Kümmerer K. (2024). Spatial trends and ecotoxic risk assessment of selected pharmaceuticals in sediments from Lake Victoria, Uganda, East Africa. Sci. Total Environ..

[6] Ngumba E., Gachanja A., Tuhkanen T. (2016). Occurrence of selected antibiotics and antiretroviral drugs in Nairobi River Basin, Kenya. Sci. Total Environ..

[7] Hossein M., Ripanda A.S. (2025). Pollution by antimicrobials and antibiotic resistance genes in East Africa: Occurrence, sources, and potential environmental implications. Toxicol. Rep..

[8] Horvat O., Kovačević Z. (2025). Human and Veterinary Medicine Collaboration: Synergistic Approach to Address Antimicrobial Resistance Through the Lens of Planetary Health. Antibiotics.

[9] Zhu Y.G., Johnson T.A., Su J.Q., Qiao M., Guo G.X., Stedtfeld R.D., Hashsham S.A., Tiedje J.M. (2013). Diverse and abundant antibiotic resistance genes in Chinese swine farms. Proc. Natl. Acad. Sci. USA.

[10] Su J.Q., Wei B., Ou-Yang W.Y., Huang F.Y., Zhao Y., Xu H.J., Zhu Y.G. (2015). Antibiotic resistome and its association with bacterial communities during sewage sludge composting. Environ. Sci. Technol..

[11] Li W.C. (2014). Occurrence, sources, and fate of pharmaceuticals in aquatic environment and soil. Environ. Pollut..

[12] Wang J., Wang S. (2016). Removal of pharmaceuticals and personal care products (PPCPs) from wastewater: A review. J. Environ. Manag..

[13] Yang Y., Ok Y.S., Kim K.H., Kwon E.E., Tsang Y.F. (2017). Occurrences and removal of pharmaceuticals and personal care products (PPCPs) in drinking water and water/sewage treatment plants: A review. Sci. Total Environ..

[14] Ortiz de García S., Pinto Pinto G., García-Encina P.A., Irusta Mata R.I. (2014). Ranking of concern, based on environmental indexes, for pharmaceutical and personal care products: An application to the Spanish case. J. Environ. Manag..

[15] Caldas S., de Oliveira Arias J.L., Rombaldi C., Mello L., Cerqueira M., Martins A., Primel E.G. (2018). Occurrence of pesticides and PPCPs in surface and drinking water in southern Brazil: Data on 4-Year Monitoring. J. Braz. Chem. Soc..

[16] Wang B.Q., Xu Z.X., Dong B. (2024). Occurrence, fate, and ecological risk of antibiotics in wastewater treatment plants in China: A review. J. Hazard. Mater..

[17] Kümmerer K. (2009). Antibiotics in the aquatic environment: A review. Chemosphere.

[18] Mlunguza N.Y., Ncube S., Mahlambi P.N., Chimuka L., Madikizela L.M. (2020). Determination of selected antiretroviral drugs in wastewater, surface water and aquatic plants using hollow fibre liquid phase microextraction and LC-MS/MS. J. Hazard. Mater..

[19] Al-Shaalan N.H., Ali I., Alothman Z., Al-Wahaibi L., Alabdulmonem H. (2019). High performance removal and simulation studies of diuron pesticide in water on MWCNTs. J. Mol. Liq..

[20] Purkait M.K., Gupta B., Das P.P. (2025). Performances of conventional treatment techniques for the treatment of pharmaceutical contaminated water: Challenges and opportunities. Advances in Chemical Engineering.

[21] Barczak M., Wierzbicka M., Borowski P. (2018). Sorption of diclofenac onto functionalized mesoporous silicas: Experimental and theoretical investigations. Microporous Mesoporous Mater..

[22] Alshehri M.A., Pugazhendhi A. (2024). Biochar for wastewater treatment: Addressing contaminants and enhancing sustainability: Challenges and solutions. J. Hazard. Mater. Adavances.

[23] Zhao Y., Qiu Y.B., Mamrol N., Ren L.F., Li X., Shao J.H., Yang X., van der Bruggen B. (2021). Membrane bioreactors for hospital wastewater treatment: Recent advancements in membranes and processes. Front. CSE.

[24] Baran W., Adamek E., Jajko M., Sobczak A. (2018). Removal of veterinary antibiotics from wastewater by electrocoagulation. Chemosphere.

[25] Bharti M., Das P.P., Purkait M.K. (2023). A review on the treatment of water and wastewater by electrocoagulation process: Advances and emerging applications. J. Environ. Chem. Eng..

[26] Hübner U., Spahr S., Lutze H., Wieland A., Rüting S., Gernjak W., Wenk J. (2024). Advanced oxidation processes for water and wastewater treatment—Guidance for systematic future research. Chem. Eng. J..

[27] Bhatia V., Malekshoar G., Dhir A., Ray A.K. (2017). Enhanced photocatalytic degradation of atenolol using graphene TiO2 composite. J. Photochem. Photobiol. A Chem..

[28] Bhattacharjee B., Ahmaruzzaman M. (2024). Photocatalytic degradation of pharmaceuticals: Insights into biochar modification and degradation mechanism. Next Mater..

[29] Cai T., Liu Y.T., Wang L.L., Dong W.Y., Zeng G.M. (2021). Recent advances in round-the-clock photocatalytic system: Mechanisms, characterization techniques and applications. J. Photochem. Photobiol. C Photochem. Rev..

[30] Brillas E., Mur E., Sauleda R., Sànchez L., Peral J., Domènech X. (1998). Aniline mineralization by AOP’s: Anodic oxidation, photocatalysis, electro-Fenton and photoelectro-Fenton processes. Appl. Catal. B Environ..

[31] Paíga P., Figueiredo S., Correia M., André M., Barbosa R., Jorge S., Delerue-Matos C. (2025). Occurrence of 97 Pharmaceuticals in Wastewater and Receiving Waters: Analytical Validation and Treatment Influence. J. Xenobiot..

[32] Tang C., Fang S.H., Yin H.L., Zhang H., Xin X., Yu B.Q., Zeng Z., Deng K.C., Zhang Y.H., Wu Z.Z. (2025). Pharmaceuticals in drinking water in suburban communities in Chengdu, China: Potential risks on the human health. Environ. Monit. Assess..

[33] Le Coadou L., Le Ménach K., Labadie P., Dévier M.H., Pardon P., Augagneur S., Budzinski H. (2017). Quality survey of natural mineral water and spring water sold in France: Monitoring of hormones, pharmaceuticals, pesticides, perfluoroalkyl substances, phthalates, and alkylphenols at the ultra-trace level. Sci. T.T.E..

[34] Farhan M., Hasani I.W., Khafaga D.S.R., Ragab W.M., Ahmed Kazi R.N., Aatif M., Muteeb G., Fahim Y.A. (2025). Enzymes as Catalysts in Industrial Biocatalysis: Advances in Engineering, Applications, and Sustainable Integration. Catalysts.

[35] Bilal M., Iqbal H.M.N. (2019). Tailoring Multipurpose Biocatalysts via Protein Engineering Approaches: A Review. Catal. Lett..

[36] Moisan J.K., Meddeb-Mouelhi F., Charbonneau D.M., Beauregard M. (2017). Impact of Salt Concentration and pH on Surface Charged Residues: Controlling Protein Association Pathways in Carboxylesterase EstGtA2. Protein Pept. Lett..

[37] Kim Y.J., Nicell J.A. (2006). Impact of reaction conditions on the laccase-catalyzed conversion of bisphenol A. Bioresour. Technol..

[38] Bhunia B., Basak B., Mandal T., Bhattacharya P., Dey A. (2013). Effect of pH and temperature on stability and kinetics of novel extracellular serine alkaline protease (70 kDa). Int. J. Biol. Macromol..

[39] Yaashikaa P.R., Keerthana Devi M., Senthil Kumar P. (2022). Advances in the application of immobilized enzyme for the remediation of hazardous pollutant: A review. Chemosphere.

[40] Duman Y.A., Tekin N. (2020). Kinetic and thermodynamic properties of purified alkaline protease from Bacillus pumilus Y7 and non-covalent immobilization to poly(vinylimidazole)/clay hydrogel. Eng. Life Sci..

[41] Ren H., Qin M.L., Zhang L., Li Z.M., Li Y.Z., He Q., Zhong J.H., Zhao D.H., Lian X.L., Jiang H.X. (2025). Modular Engineering of a Synthetic Biology-Based Platform for Sustainable Bioremediation of Residual Antibiotics in Aquatic Environments. Engineering.

[42] Wu A., Sha F., Su S., Farha O.K. (2024). Recyclable Enzymatic Hydrolysis with Metal-Organic Framework Stabilized Humicola insolens Cutinase (HiC) for Potential PET Upcycling. Chem. Bio. Eng..

[43] Noman E., Al-Gheethi A.A., Talip B.A., Mohamed R., Kassim A.H. (2020). Oxidative Enzymes from Newly Local Strain Aspergillus iizukae EAN605 Using Pumpkin Peels as a Production Substrate: Optimized Production, Characterization, Application and Techno-Economic Analysis.

[44] Wu D., Khan S., Zhang S.J., Wang H., Chen W., Wang S.Q. (2025). Self-assembled Immobilization and Metal-Polyphenol Network Encapsulation of β-Galactosidase on T4 phage for Enhanced Biocatalytic Performance. Appl. Biochem. Biotechnol..

[45] Karam E.A., Abdel Wahab W.A., Saleh S.A.A., Hassan M.E., Kansoh A.L., Esawy M.A. (2017). Production, immobilization and thermodynamic studies of free and immobilized Aspergillus awamori amylase. Int. J. Biol. Macromol..

[46] Ghaedmohammadi S., Nooraei S., Ahrari F., Moosavi F., Mohammadi M., Ahmadian G. (2025). Thermostable and recyclable Candida antarctica lipase B immobilized on Bacillus subtilis using sortase technology. Microbiol. Spectr..

[47] Joss A., Zabczynski S., Göbel A., Hoffmann B., Löffler D., McArdell C.S., Ternes T.A., Thomsen A., Siegrist H. (2006). Biological degradation of pharmaceuticals in municipal wastewater treatment: Proposing a classification scheme. Water Res..

[48] Hu M., He W.S., Jiang R., Zhang Y., Wang X.H. (2025). Bottom-up artificial construction of the M7-Z4 bacterial model enhanced sulfamethazine mineralization: Metagenomic analysis combined with bacterial isolation techniques. J. Hazard. Mater..

[49] Baena-Nogueras R.M., González-Mazo E., Lara-Martín P.A. (2017). Degradation kinetics of pharmaceuticals and personal care products in surface waters: Photolysis vs biodegradation. Sci. Total Environ..

[50] Wang L.J., Wang X.Y., Wu H., Wang H.X., Lu Z.M. (2024). Interspecies synergistic interactions mediated by cofactor exchange enhance stress tolerance by inducing biofilm formation. mSystems.

[51] Chen X.Y., Zhang X., Zhao X.Y., Xi B.D., Lu Y. (2025). Microbial co-metabolism in the degradation of emerging organic pollutants. J. Environ. Sci..

[52] Dong T.J., Zhang L., Yang J.C., Hao S.W., Peng Y.Z. (2026). Photocatalysis-fueled algae-bacteria mutualism enables P450 enzyme-induced nitrite-free anammox for sustainable nitrogen removal. Water Res..

[53] Cui L., Chen J., Yan Y., Fei Q., Ma Y., Wang Q. (2024). Development of oriented microbial consortium-based compound enzyme strengthens food waste hydrolysis and antibiotic resistance genes removal: Deciphering of performance, metabolic pathways and microbial communities. Environ. Res..

[54] Zheng D., Zheng Y.L., Tan J.J., Zhang Z.J., Huang H., Chen Y. (2024). Co-immobilization of whole cells and enzymes by covalent organic framework for biocatalysis process intensification. Nat. Commun..

[55] Chen Z.M., Fan Q.F., Wang J.H., Zhang J.Y., Zhang Y.H., Tan T.W., Lv Y.Q. (2025). Designing a mesoporous cascade reactor for enhanced enzymatic performance. Bioresour. Technol..

[56] Xue K., Bai Z.H., Fordour E., Guo S.Q., Zhou Y.X., Yang Y.K., Liu X.X., Li Y., Liu C.L. (2024). Bacterial surface display of PETase mutants and MHETase for an efficient dual-enzyme cascade catalysis. Bioresour. Technol..

[57] Huang L., Jin Y., Zhou D.H., Liu L.X., Huang S.K., Zhao Y.Q., Chen Y.C. (2022). A Review of the Role of Extracellular Polymeric Substances (EPS) in Wastewater Treatment Systems. Int. J. Environ. Res. Public Health.

[58] Li D.Y., Wu Y.S., Liang D.B., Li J.R., Xie C.F., Zhu Y.H., Liu T.T., Du L.Z., Yao X.R., Liu W.Q. (2025). Multi-omics insights into ROS-mediated molecular responses of extracellular polymeric substances in aerobic granular sludge under micro/nanoplastic stress. Chem. Eng. J..

[59] Zou S.P., Zhang B., Han Y.Y., Liu J.L., Zhao K., Xue Y.P., Zheng Y.G. (2024). Design of a cofactor self-sufficient whole-cell biocatalyst for enzymatic asymmetric reduction via engineered metabolic pathways and multi-enzyme cascade. Biotechnol. J..

[60] dos Santos K.P., Rios N.S., Labus K., Gonçalves L.R.B. (2022). Co-immobilization of lipase and laccase on agarose-based supports via layer-by-layer strategy: Effect of diffusional limitations. Biochem. Eng. J..

[61] Yang W.H., Sun H., Cui Z.H., Chen L., Ji Y., Lu F.P., Liu Y.H. (2024). Spatially sequential co-immobilization of phosphorylases in tiny environments and its application in the synthesis of glucosyl glycerol. Int. J. Biol. Macromol..

[62] Sun J.H., Yan M.J., Tao G.D., Su R.B., Xiao X.M., Wu Q.S., Chen F., Wu X.L., Lin H.J. (2025). A single-atom manganese nanozyme mediated membrane reactor for water decontamination. Water Res..

[63] Wang Z., Sun Y. (2022). A hybrid nanobiocatalyst with in situ encapsulated enzyme and exsolved Co nanoclusters for complete chemoenzymatic conversion of methyl parathion to 4-aminophenol. J. Hazard. Mater..

[64] Du J., Dang X., Zhao H. (2025). Photo-enzyme cascade catalysis treatment of bisphenol A in water: Synergistic hydroxylation pathway for mineralization and detoxification. J. Hazard. Mater..

[65] Wang X.H., Wang G.J., Xu X.L., Xu T., He Y.W., Zhang X.Y., Yang S.H., Fan J.H., An X.F. (2025). In-situ assembled biochar-yeast hybrid for tetracycline detoxification: Unparalleled robustness, toxicity assessment, and multi-omics analysis. Chem. Eng. J..

[66] Oh S., Nguyen H.T., Obayomi K.S., Siddiqui S.I. (2025). Assessing the environmental risk potential of transformation byproducts formed during fungal enzymatic treatment of a pharmaceutical mixture. J. Ind. Eng. Chem..

[67] Fu X.J., Fei Q.R., Zhang X.J., Li N., Zhang L., Zhou Y. (2024). Two different types of hydrolases co-degrade ochratoxin A in a highly efficient degradation strain *Lysobacter* sp. CW239. J. Hazard. Mater..

[68] Jiang Y.H., Huang X., Yu Y.X. (1993). A Comparative Study on Several Carriers of Immobilized Cells. Environ. Sci. Resour. Util..

[69] Li P., Chen Q.S., Wang T.C., Vermeulen N.A., Mehdi B.L., Dohnalkova A., Browning N.D., Shen D.K., Anderson R., Gómez-Gualdrón D.A. (2018). Hierarchically Engineered Mesoporous Metal-Organic Frameworks toward Cell-free Immobilized Enzyme Systems. Chem.

[70] Chen T.P., Wang S.M., Niu H.Q., Yang G.J., Wang S.N., Wang Y.Q., Zhou C.W., Yu B., Yang P.P., Sun W.J. (2023). Biofilm-Based Biocatalysis for Galactooligosaccharides Production by the Surface Display of β-Galactosidase in Pichia pastoris. Int. J. Mol. Sci..

[71] Sooriyakumar P., Bolan N., Kumar M., Singh L., Yu Y., Li Y., Weralupitiya C., Vithanage M., Ramanayaka S., Sarkar B. (2022). Biofilm formation and its implications on the properties and fate of microplastics in aquatic environments: A review. J. Hazard. Mater. Adv..

[72] Flemming H.C., van Hullebusch E.D., Neu T.R., Nielsen P.H., Seviour T., Stoodley P., Wingender J., Wuertz S. (2023). The biofilm matrix: Multitasking in a shared space. Nat. Rev. Microbiol..

[73] Carr C.M., Harkova L.G., McCarthy R.R. (2025). Engineering biology approaches to modulate bacterial biofilms. Trends Biotechnol..

[74] Özkul G., Şahin Kehribar E., Ahan R.E., Şeker U.Ö.Ş. (2024). An antibiotic-degrading engineered biofilm platform to combat environmental antibiotic resistance. ACS Biomater. Sci. Eng..

[75] Zhang X.E., Liu C.L., Dai J.B., Yuan Y.J., Gao C.X., Feng Y., Wu B., Wei P., You C., Wang X.W. (2023). Enabling technology and core theory of synthetic biology. Sci. China Life Sci..

[76] Liu W., Sun W.J., Liang C.C., Chen T.P., Zhuang W., Liu D., Chen Y., Ying H.J. (2025). Escherichia coli Surface Display: Advances and Applications in Biocatalysis. ACS Synth. Biol..

[77] Taw M.N., Li M.J., Kim D., Rocco M.A., Waraho-Zhmayev D., DeLisa M.P. (2021). Engineering a supersecreting strain of Escherichia coli by directed coevolution of the multiprotein TAT translocation machinery. ACS Synth. Biol..

[78] Helvig C., Kariyawasam T., Vriens B., Petkovich M. (2025). Genetically engineered bacteria and microalgae expressing a mutant of cytochrome P450 BM3 for efficient Diuron degradation in wastewater treatment. Appl. Environ. Microbiol..

[79] Doron L., Kerfeld C.A. (2024). Bacterial microcompartments as a next-generation metabolic engineering tool: Utilizing nature’s solution for confining challenging catabolic pathways. Biochem. Soc. Trans..

[80] Amin M.F., Rahman M.S. (2026). A critical review of pharmaceutical pollutants in soil and air: Ecotoxicological impacts on animal, plant and microbial communities—Health hazards and waste management. J. Hazard. Mater. Adv..

[81] Dzionek A., Nowak A., Wojcieszyńska D., Potocka I., Smułek W., Guzik U. (2024). Decomposition of non-steroidal anti-inflammatory drugs by activated sludge supported by biopreparation in sequencing batch reactor. Bioresour. Technol..

[82] Bhardwaj P., Sharma S., Khatri M., Singh G., Arya S.K. (2023). Eradication of ibuprofen and diclofenac via in situ synthesized and immobilized bacterial laccase to Cu-based metal organic framework. J. Water Process Eng..

[83] Peng H.X., He Y.Z., Li T.Y., Peng X.X. (2024). Acyclovir contamination in environment: Occurrence, transformation, toxicity, risk, and evaluation as a pharmaceutical indicator. Sci. Total Environ..

[84] Karungamye P., Rugaika A., Mtei K., Machunda R. (2022). The pharmaceutical disposal practices and environmental contamination: A review in East African countries. HydroResearch.

[85] Bernardes M.F.F., Pazin M., Pereira L.C., Dorta D.J. (2015). Impact of Pesticides on Environmental and Human Health.

[86] Daughton C.G. (2007). Pharmaceuticals in the environment: Sources and their management. Comprehensive Analytical Chemistry.

[87] Dube S. (2025). Green and sustainable pharmacology: Integrating environmental responsibility into drug discovery, development, and practice. IP Int. J. Compr. Adv. Pharmacol..

[88] Akhrimenko V., Kümmerer K., Malato S., Lertxundi U., Orive G. (2025). Designing at-source and end-of-pipe biotechnologies to tackle pharmaceutical pollution. Trends Biotechnol..

[89] Parker G., Miller F.A. (2024). Tackling Pharmaceutical Pollution Along the Product Lifecycle: Roles and Responsibilities for Producers, Regulators and Prescribers. Pharmacy.

[90] Caban M., Stepnowski P. (2021). How to decrease pharmaceuticals in the environment? A review. Environ. Chem. Lett..

[91] Medhi B., Sewal R. (2012). Ecopharmacovigilance: An issue urgently to be addressed. Indian J. Pharmacol..

[92] Dzidzornu E., Cherian J.J., D’souza J., Pandit J. (2023). Ecopharmacovigilance: A review of cause, impact, and remedies. Med. Writ..

[93] WHO (2023). One Health. https://www.who.int/news-room/fact-sheets/detail/one-health.

[94] EU (2020). European Parliament Resolution on a Strategic Approach to Pharmaceuticals in the Environment. https://www.europarl.europa.eu/doceo/document/TA-9-2020-0226_EN.html.

[95] OECD (2019). Pharmaceutical Residues in Freshwater: Hazards and Policy Responses.

[96] Stefanache A., Marcinschi A., Marin G.A., Mitran A.M., Lungu I.I., Miftode A.M., Crivoi F., Lacatusu D., Baican M., Cioanca O. (2025). Green Chemistry Approaches in Pharmaceutical Synthesis: Sustainable Methods for Drug Development. AppliedChem.

[97] Chacón M., Alvarez-Gonzalez G., Gosalvitr P., Berepiki A., Fisher K., Cuéllar-Franca R., Dixon N. (2025). Complex waste stream valorization through combined enzymatic hydrolysis and catabolic assimilation by Pseudomonas putida. Trends Biotechnol..

[98] He D., Wen Y.J., Wei S.Z., Li S.K., Liu L.D., Wu J.M., Zhou Z., Zhou N., Liu H.M., Zhou Z.H. (2025). Conversation of pesticide residues into ammonium nitrogen (NH_4_^+^-N) through AOPs and its fertilization effect on lettuce growth. Biochar.

[99] Jiang C., Meng Z. (2025). pH and CO_2_/N_2_ dual responsive Pickering emulsion stabilized by shellac nanoparticle-enzyme conjugates for synthesis of phytosterol esters. Food Chem..

[100] Wang P.C., Yang X.W., Lin B.X., Huang J.Z., Tao Y. (2017). Cofactor self-sufficient whole-cell biocatalysts for the production of 2-phenylethanol. Metab. Eng..

[101] Mehta N. (2026). The synthetic microbial ecosystem: Rational design, dynamic control, and translational impact of consortia for sustainable waste valorization. ChemRxiv.

[102] Ishak S.N.H., Mat Saad A.H., Latip W., Rahman R.A.R.N.Z., Salleh A.B., Ahmad Kamarudin N.H., Leow A.T.C., Mohamad Ali M.S. (2025). Enhancing industrial biocatalyst performance and cost-efficiency through adsorption-based enzyme immobilization: A review. Int. J. Biol. Macromol..

[103] Fisher S.W., Briggs J.D. (1988). Environmental and ecological problems in the introduction of alien microorganisms in the soil. Agric. Ecosyst. Environ..

[104] Arnold B.J., Huang I.-T., Hanage W.P. (2022). Horizontal gene transfer and adaptive evolution in bacteria. Nat. Rev. Microbiol..

[105] Kennedy G.J., Bowman M.J., Ascherl K.L., Nichols N.N., Saha B.C. (2024). Biomass Demineralization and Pretreatment Strategies to Reduce Inhibitor Concentrations in Itaconic Acid Fermentation by *Aspergillus terreus*. Biomass.

[106] Kim H.S., Lee J.Y., Cho Y.J., Kim H.S., Kim H.Y., Sung B.H. (2025). Governing Synthetic Biology: A Co-Evolutionary Framework for Sustainable Innovation. J. Microbiol. Biotechnol..

[107] Salzano D., Shannon B., Grierson C., Marucci L., Savery N.J., di Bernardo M. (2024). In-vivo distributed multicellular control of gene expression in microbial consortia. bioRxiv.

[108] Lázaro H.M., Otero-Muras I., Carbonell P. (2025). BiosInt: Biosensor-based smart design of pathway dynamic regulation for industrial biomanufacturing. bioRxiv.

[109] Hsieh Y.L. (2026). Computational Integration of Multi-Omics and Phenotype Data Into Genome-Scale Metabolic Models.

[110] Molina Ortiz J.P., McClure D.D., Holmes A., Rice S.A., Read M.N., Shanahan E.R. (2024). Genome scale metabolic modelling of human gut microbes to inform rational community design. bioRxiv.

[111] Su C., Cui H.T., Wang W.W., Liu Y., Cheng Z.Y., Wang C., Yang M.Q., Qu L.W., Li Y., Cai Y.J. (2025). Bioremediation of complex organic pollutants by engineered Vibrio natriegens. Nature.

[112] Madani A., Krause B., Greene E.R., Subramanian S., Mohr B.P., Holton J.M., Olmos J.L., Xiong C.M., Sun Z.Z., Socher R. (2023). Large language models generate functional protein sequences across diverse families. Nat. Biotechnol..

[113] Chauhan S., Singh I., Singh M., Sominder A. (2026). Biotechnological Advancements in Active Pharmaceutical Ingredient Removal: Sustainable Solutions for Pharmaceutical Wastewater Treatment. Curr. Green Chem..

[114] Sesay F., Sesay R.E.V., Kamara M., Li X.S., Niu C.X. (2025). Biodegradation of pharmaceutical contaminants in wastewater using microbial consortia: Mechanisms, applications, and challenges. J. Environ. Manag..

